# Memory engram stability and flexibility

**DOI:** 10.1038/s41386-024-01979-z

**Published:** 2024-09-18

**Authors:** Yosif Zaki, Denise J. Cai

**Affiliations:** Nash Department of Neuroscience, Icahn School of Medicine at Mount Sinai, New York, NY USA

**Keywords:** Long-term memory, Neuroscience

## Abstract

Many studies have shown that memories are encoded in sparse neural ensembles distributed across the brain. During the post-encoding period, often during sleep, many of the cells that were active during encoding are reactivated, supporting consolidation of this memory. During memory recall, many of the same cells that were active during encoding and reactivated during consolidation are reactivated during recall. These ensembles of cells have been referred to as the memory engram cells, stably representing a specific memory. However, recent studies question the rigidity of the “stable memory engram.” Here we review the past literature of how episodic-like memories are encoded, consolidated, and recalled. We also highlight more recent studies (as well as some older literature) that suggest that these stable memories and their representations are much more dynamic and flexible than previously thought. We highlight some of these processes, including memory updating, reconsolidation, forgetting, schema learning, memory-linking, and representational drift.

## Introduction

Memory is a fundamental function of many living systems. It allows us to retain a history of our past experiences—our past interactions with the world and the consequences of those interactions. And this history guides our future interactions with the world; the past tells us what should be avoided and what might be worth our while. Yet, the world is an ever-changing environment, and humans—among many animals—have a remarkable ability to flexibly regulate our memories. Memories are constantly being formed as we have new experiences, and we filter which of these experiences should be stored, which past memories might now be obsolete and thus should be updated, which memories should be forgotten; and we use our memories to inform the way that we learn in the future. This rich memory system is central to survival, and it arguably defines our life experience. As Jorge Luis Borges wrote, “We are our memory, we are this chimerical museum of shifting forms, this heap of broken mirrors” [1].

The nineteenth and twentieth centuries saw significant advances in the research of memory which set the stage for the study of memory today. In 1904, Richard Semon published a book seeking to formalize the definition of memory. What is a memory? In this work, he defined two terms that have endured until today. The first, the “engram,” was defined as the “enduring though primarily latent modification in the irritable substance produced by a stimulus.” To paraphrase, this term was meant to represent the lasting changes in an organism after an experience which store a memory. The second term, “ecphory,” was defined as the process of “awaken[ing] the mnemic trace or engram out of its latent state into one of manifested activity.” In other words, memory recall [2, 3].

Much of the twentieth century was concerned with discovering how the brain stores and recalls memories [4]. In the reductionist manner that is common in biology, rather than studying memory in its full everyday richness, the field had largely studied the storage, maintenance, and recall of single experiences. This approach contributed a vast wealth of knowledge toward our understanding of the neurobiological basis of memory. Famously, Karl Lashley was among the first to study the neural basis of memory, and his goal was to find the locus of memory in the brain. His approach was to systematically remove different parts of the brain (of rats and monkeys) and measure any resulting impairment in memory. Lashley did not find that removal of any one part of the brain abolished an animal’s capacity to learn. Rather, he found that the amount of resected brain was related to the degree of memory impairment. Summarizing decades worth of efforts, Lashley concluded that there was no locus of memory in the brain [5]. Shortly thereafter, a historic clinical case study gave a hint about the neural basis of memory—in particular, of declarative memory—and it changed the field of memory research thereafter. Patient H.M. was a teenager who was suffering from epilepsy. In 1953, he underwent an experimental surgery to have both hemispheres of his medial temporal lobe resected. This operation did indeed decrease his seizures; however, he also unexpectedly had a dramatic loss in the ability to form new episodic memories—memories for everyday personal experiences [6]. In particular, he had deficits in the ability to recall recently formed episodic memories; however, he was still able to recall older episodic memories and was able to form new procedural, motor skill-based memories. These findings hinted at the possibility that different parts of the brain might be involved in different forms of memory and in the short versus long-term storage of memories [7]. Patient H.M. was studied in great detail throughout his life after his surgery and he has contributed immensely to our understanding of memory. This case study highlighted that there may indeed be a locus in the brain where episodic memories are formed, and decades of work since then have indeed confirmed the importance of areas in the medial temporal lobe, such as the hippocampus and entorhinal cortex, in the formation, maintenance, updating, and recall of memories [8].

Today, the tools available to observe and manipulate the brain in rodents are remarkable, and they have provided us with immense detail about the neurobiological bases of memory. Below we review the current understanding of episodic-like memory in the brain, from its formation and consolidation to its recall and updating. To gain a full understanding of memory, we need to study not only how a single memory is represented in the brain, but also how multiple memories interact. Thus, we highlight efforts that have been taken to understand how memories encoded across time are integrated or segregated. There are many questions regarding the neurobiology of memory that remain, and we highlight some potential future directions that may be taken. The study of basic mechanisms of memory will pave the way for understanding not only how memory functions in everyday settings, but also how memory goes awry in psychiatric conditions. Many psychiatric conditions have disruptions in memory processes among their symptomatology [9], highlighting the need for understanding the neural mechanisms underlying neurotypical memory. Ultimately, we believe that understanding the basic neurobiology of memory will be beneficial not only for the fundamental understanding of memory but may also inform clinical intervention [10–12].

## Memory encoding

**Fig. 1:**
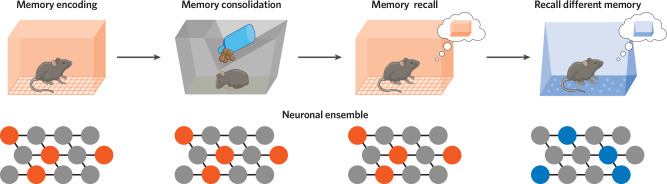
A canonical view of the stable memory engram. Many studies have shown that memories are encoded in sparse neural ensembles distributed across the brain. After encoding, studies have shown that many of those same cells are reactivated during an offline period (e.g., sleep), stabilizing and consolidating the neural ensemble representing the memory. During recall, many of the same cells are reactivated, which have been thought to underlie the stable memory representation (i.e., the memory engram). The brain can store distinct memories through different ensembles of cells and different neural activity patterns.

The formation of a memory begins during an experience. When an animal has a novel experience, such as exploration of a novel environment, subpopulations of neurons are active across the brain (Fig. 1). On a cellular level, these high levels of neuronal activity lead to subsequently high levels of intracellular calcium inside these neurons. Calcium drives a host of downstream signaling via calcium-dependent proteins in neurons and this downstream signaling drives short-term biochemical plasticity as well as long-term structural plasticity [13]. This increase in neuronal activity also elicits transcription of plasticity-related genes, including a class of genes termed immediate early genes (IEGs) whose transcriptional activity is necessary for learning [14]. Since these genes are transcribed in response to high neuronal activity, recent genetic tools have been aimed at labeling the neurons which express these genes to gain insight into which groups of neurons were active during learning and underwent subsequent gene transcriptional changes relevant for plasticity [15, 16]. These results have demonstrated that a critical feature of learning is the gene signaling changes that occur during an experience to drive long-term synaptic plasticity in neurons active during learning and specifically between pre-synaptic and post-synaptic neurons active during learning [17]. Notably, not all neuronal activity leads to expression of IEGs, and the relationship between a neuron’s electrical activity and IEG expression is currently of great interest. For example, brief neuronal activation leads to expression of a smaller set of activity-regulated genes compared with sustained neuronal activation [18]. Another study electrophysiologically recorded hippocampal neurons while mice explored a novel context and identified which of the recorded neurons expressed cFos (an IEG) during the context exploration. cFos-positive cells were more likely to exhibit burst-like firing than cFos-negative cells and cFos-positive cells were less likely to fire reliably in a specific location in the environment (i.e., they had less stable place fields) [19]. This suggests that cells that exhibit burst-like activity will go on to express the IEG cFos. One final study found, in contrast, that cells that go on to express cFos exhibit highly stable place fields and form ensembles that are more likely to fire together [20]. Thus, the relationship between neuronal electrical activity, IEG expression, and the relationship to behavior remain incompletely understood.

Some work has suggested that the neurons that go on to be highly active during learning of a novel experience are already privileged to become highly active even prior to that experience [21]. For example, it has been shown that neurons with high excitability just prior to learning are more likely to become part of the memory of that event [22], and that artificially increasing expression of the transcription factor, CREB, in a subset of neurons in the amygdala makes those neurons more likely to then become part of the memory [23]. More recent work has shown that not only are those neurons which are destined to become part of a memory trace highly active prior to learning, but groups of hippocampal neurons which are destined to become memory-relevant cells after learning are also more likely to have correlated activities just prior to learning as well [24]. It has also been shown that neurons in the hippocampus that fire in sequential order during learning also exhibit these temporally ordered patterns of activity prior to learning as well, termed “preplay” [25]; however, the existence of preplay has been debated [26]. Two recent studies have shown that activity patterns in the brain can predict future neuronal responses to stimuli in the visual cortex [27] and in the hippocampus [28]. These studies have collectively suggested that experience does not necessarily imprint de novo patterns of activity onto the brain. Instead, the brain has ongoing patterns of activity that are evolving across time, and when experiences occur, these patterns of neuronal activity gain meaning and are reinforced [29]. It remains unclear how long before an experience these pre-configured patterns of neuronal activity are detectable, and whether these timescales vary across brain regions. For example, most studies of pre-configured activity patterns prior to learning have been studied in the hippocampus, where it has been observed that neuronal activity within minutes to hours of an experience is predictive of the cells active during that experience [24, 25, 28]. However, it has also been observed that spontaneous hippocampal activity up to two days prior to an experience is predictive of activity during learning two days later [30]. Whether hippocampal activity is predictive of future activity beyond two days has yet to be shown. In the visual cortex, stimulus-evoked ensemble activity gradually drifts across days [27]. It will be interesting for future studies to explore how long before an experience pre-configured ensemble activity is detectable and whether and how this varies in different brain circuits.

Finally, the coordinated timing of activity of groups of neurons (or, neuronal ensembles) is important for driving learning [31]. When, for example, an experience is paired with a salient outcome, the temporal order of neuronal activity among a neuronal population is important for driving this process [32–34]. In classical conditioning where a neutral conditioned stimulus (CS) is paired with a valenced unconditioned stimulus (US) [35], the CS must typically occur close in time to the US for classical conditioning between the stimuli to take place. For example, as the temporal delay increases between the CS and US, the conditioned response in response to the CS decreases—termed trace conditioning [36]. This temporal delay typically occurs on the order of seconds to minutes. One neurobiological account to explain associative learning is based on coincident activity among stimulus-responsive neurons; when presynaptic and postsynaptic neurons fire close in time to one another, this can drive long-term potentiation (LTP) between these neurons such that they are more likely to fire together in the future [31, 37]. For decades, it was believed that pre- and post-synaptic neurons must be active within dozens of milliseconds of each other to strengthen the connection between them. This was termed Hebbian plasticity after Donald Hebb postulated that repeated and coincident activity between pre- and post-synaptic neurons may drive their strengthening [38]. Recently, however, Bittner et al. were studying how place cells—hippocampal neurons that fire when an animal is in specific locations in an environment [39]—form, and they discovered a novel form of plasticity that strengthens inputs to a post-synaptic neuron that were active across several seconds. They found that strong depolarization of CA1 pyramidal neuron dendrites (i.e., plateau potential) drove CA1 neurons to strengthen inputs that occurred within several seconds of the plateau potential, and that a single plateau potential was sufficient to drive the rapid formation of place fields—termed behavioral time-scale plasticity (BTSP) [40]. BTSP has been discovered in hippocampal CA1; however, whether this form of synaptic plasticity occurs in other areas of the brain remains an unexplored and exciting possibility. A recent preprint suggests that BTSP may occur among excitatory recurrent connections in hippocampal CA3 as well [41]. Whether BTSP occurs outside of the hippocampus, and whether BTSP occurs during different forms of learning beyond the formation of place fields, remains to be investigated. The discovery of BTSP suggested that neuronal activity that occurs across as long as seconds could be linked, beyond the milliseconds delay necessary for LTP. How stimuli that occur across longer periods of time, such as hours to days, become associated is comparatively less well understood. LTP on the order of milliseconds and BTSP on the order of seconds is insufficient to explain how animals are capable of associating information across much longer spans of time. We highlight some recent efforts which have investigated learning on these longer timescales in the later section *Memory Updating*. Collectively, the fine-tuned temporal activity among neurons regulates the strengthening and weakening of synaptic connections relevant to drive learning.

## Memory consolidation

After an experience initially drives robust neuronal activity to initiate the formation of a memory, a process of memory consolidation occurs for the hours thereafter whereby the experience is stored in the brain for future retrieval. This process of long-term storage requires that long-term changes are made in the brain to store the information related to the experience—the engram if you will. Many studies on memory consolidation in the brain have suggested that de novo protein synthesis in the hours after learning was necessary for learning to occur. This was originally concluded after administration of protein synthesis inhibitors shortly after learning disrupted memory for the event [42]. After several studies demonstrated similar disruptions in memory after administration of a protein synthesis inhibitor, a debate began about whether the disruptions in memory were due to unintended side effects of the protein synthesis inhibitors [43, 44]. To this day, it remains unclear under what conditions de novo protein synthesis is required for memory consolidation; however, a recent tool was developed to disrupt protein synthesis in vivo in a cell type-specific manner, and this manipulation indeed showed that disruption of protein synthesis after learning disrupted long-term memory [45]. Protein synthesis-dependent memory consolidation is thought to occur for around 6 h following an experience. Originally, it was observed that after a learning event, if a second learning event is imposed shortly thereafter, it interferes with the first event. This suggested that perhaps the period after learning is a sensitive period during which experiences must be stored in memory [46, 47]. Since then, a notable study showed that manipulation of protein synthesis within 6 h after learning disrupts subsequent memory, while disruption of protein synthesis thereafter does not, which suggests that there is a critical window after an experience during which the experience is solidified in memory [48]. The synaptic tag and capture hypothesis (STC) has suggested that a novel experience can drive the accumulation of plasticity-related proteins at specific synapses, which “tag” those synapses, even if they do not directly potentiate them. As a result, subsequent experience can then produce potentiation at already tagged synapses, without the need for novel protein synthesis [49]. Once a memory is formed and stored, it is not immutable. In fact, subsequent experiences can modify past memories in a multitude of ways [50, 51], a topic that we will revisit in a later section.

On a longer timescale, memories are thought to also consolidate across weeks to years, and this distinct mechanism has been termed systems consolidation. This framework states that memories are rapidly formed through synaptic plasticity in the hippocampus, and this memory is transferred to cortical areas across weeks. This transformation is thought to be accompanied by memories becoming less specific and more gist-like [52]. Some studies suggest that the hippocampus is necessary for initial learning; however, it becomes dispensable for memory recall after weeks of systems consolidation and this role is instead transferred to the cortex [53], although this framework has been contentious [54]. Specifically, it has been unclear the degree to which the hippocampus becomes dispensable for remote memory recall (weeks after learning), and the degree to which the cortex is involved in recent memory recall (days after learning). For example, one study showed that during learning, cortical neurons undergo plasticity to form “silent engrams,” which are not involved to drive memory recall at recent time points (i.e., days after learning), but which become active to drive memory recall at remote time points (i.e., weeks after learning). If cortical activity is blocked during learning, there is no apparent effect on memory recall shortly after learning but the impairment in memory recall emerges after weeks [55]. This result suggests that the cortex plays a role in promoting long-term memory formation at the time of learning, though this role becomes apparent only weeks after learning. One recent review has claimed that it may be time to abandon the term “memory consolidation” with a term that acknowledges that memories are not static and that rather, they are dynamically updated across time and experience [56]. We will return to the idea that memories are dynamic in a later section. Collectively, this work on memory consolidation demonstrates that memories are stored across multiple co-occurring timescales.

When animals undergo a spatially sequential experience (such as running along a maze in a repeated order), hippocampal neurons fire in particular spatial locations to produce a map of the environment [39]. A remarkable discovery was that after the experience when animals are resting (i.e., during “offline periods”), they reactivate the patterns of neuronal activity that were previously active during the experience (Fig. 1) [57–60]. These “reactivation events” or “replay events” are not confined to the hippocampus—for example, they have also been observed in cortical areas [61, 62], although hippocampal reactivation after experience has been the most well-characterized form of reactivation after an experience. Hippocampal replay events are frequently nested within sharp wave ripples, which are brief oscillatory events detected in the hippocampal local field potential. They typically consist of a large amplitude deflection in the radiatum of hippocampal CA1 accompanied by a brief high-frequency oscillation (100–200 Hz) in the pyramidal layer of CA1, and these events were thought to help store the memory for the temporally structured event [63, 64]. Recent studies have asked whether these sharp wave ripples are causally relevant for driving memory consolidation. Indeed, disrupting sharp wave ripples disrupts memory consolidation [65], inhibiting hippocampal replay disrupts memory consolidation [66], and elongating sharp wave ripples enhances memory [67]. Hippocampal replay has typically been measured within the first hour or a few hours following a salient experience [58, 60]. One study demonstrated that hippocampal replay persisted after an experience for up to 10 h [68], making it unclear how long hippocampal replay may persist for after an experience. In connection with the mechanisms of consolidation, it remains unknown how hippocampal replay is related to cellular or systems memory consolidation, although this is a question of great interest in the field [69]. It is known that hippocampal replay during sharp wave ripples coincides with the occurrence of spindles in the cortex [70–72]. This coordinated activity between the hippocampus and cortex allows for memory-relevant neuronal activity to be broadcast across the cortex in close temporal proximity, perhaps allowing for subsequent synaptic plasticity in the cortex to bind together the constituents of a memory. Future studies aimed at bridging hippocampal and cortical reactivation during offline periods with long-term synaptic plasticity changes for systems consolidation will be an important direction to explore [63]. In sum, growing evidence suggests that temporally structured neuronal activity patterns during offline periods after learning are critical for proper memory consolidation.

## Memory recall

When an animal recalls a memory that was previously formed and consolidated, the neural representation of that memory is activated in the brain. A central question in the field of memory has been, what is the stable neural representation of a memory? One landmark study demonstrated that the neurons in the amygdala that are highly active during learning of an experience are also highly active during recall of that experience, and the degree of reactivation of the learning-active cells during recall is correlated with the behavioral strength of the memory (Fig. 1) [73]. This was evidence that memory could at least in part be represented by reactivation of the neurons that were highly active during learning. Since then, significant efforts have been made to exhaustively test this hypothesis, with many studies converging on similar conclusions. For example, one study genetically labeled the hippocampal neurons active during fear conditioning and inhibited those neurons thereafter during recall. They found that inhibition of the learning-active neurons disrupted memory recall [74]. Another study similarly labeled hippocampal neurons active during fear conditioning, but later excited these neurons in a different environment where the mice had not had any negative experience. They found that artificially stimulating these learning-active neurons in the neutral context was sufficient to drive artificial memory recall, such that the animals displayed fear-related behavior in the otherwise neutral context [75].

## Memory updating

### Extinction learning and reconsolidation

Of course, memories are not static once formed. They are dynamically updated as animals have novel experiences throughout their lifetime. Memories can be updated in myriad ways. One form of memory updating that has been extensively studied is extinction learning, in particular fear extinction learning. Fear extinction occurs after fear conditioning between a conditioned stimulus (e.g., an environment) and unconditioned stimulus (e.g., an electric shock) has occurred and it refers to the extended exposure of an animal to the conditioned stimulus in the absence of the unconditioned stimulus, which detaches the association between the conditioned and unconditioned stimuli [76]. The neural basis of extinction learning has been extensively studied. Some studies have shown that the synaptic plasticity changes that occur during initial fear conditioning are undone during extinction learning [77–79]. In other words, this work suggests that fear extinction represents “unlearning” of the original fear conditioning memory. Other literature suggests that fear extinction learning represents a second form of learning which inhibits the original fear memory, but without explicitly dismantling the original fear memory representation [80–82]. Whether extinction learning represents unlearning of the original fear memory or new learning of an extinction memory is still debated, and one recent idea that was proposed suggests that perhaps extinction is represented by a mixed mechanism of both unlearning and new learning [83]. Considering that extinguished fear memories are still prone to fear relapse suggests that the original fear memory still exists in the brain, albeit perhaps in a latent state [84]. Relapsed fear memory representations do not fully revert to their original fear memory state even after relapse, consistent with a mixed mechanism [85].

Another form of memory updating is memory reconsolidation. Memory reconsolidation refers to a state shortly after memory recall, during which memories are malleable and can be updated. A breakthrough study that set the stage for the memory reconsolidation field was conducted by Karim Nader and colleagues in 2000 [86]. In this study, the authors administered anisomycin, a protein synthesis inhibitor into the amygdala of rats following fear memory recall, a day after the fear memory was initially consolidated and thought to be stably stored. They found that anisomycin administration immediately after memory recall disrupted subsequent memory, suggesting that memory recall put the memory into a malleable state after which it could be updated [87]. This ignited a field aimed at understanding how memories are dynamically updated through reconsolidation [88]. It has been postulated that memory consolidation and reconsolidation represent the same phenomenon, whereby memories are constantly updated as animals have novel experiences [50]. An exciting application of the reconsolidation framework has been to harness reconsolidation mechanisms to update highly aversive memories in humans by modifying them to be paired with non-fearful information during memory recall [89].

### Forgetting

While many salient memories that we store last a lifetime, many memories that we once formed are later forgotten. The purpose or function of forgetting is not well understood; however, a leading idea is that forgetting prevents “oversaturation” of unnecessary information, which then permits efficient learning [90]. The neural processes underlying forgetting have been studied in some detail, with one leading theory positing that the synaptic alterations that were originally made to store the memory are undone, which leads the memory to be inaccessible for recall [91]. An exciting recent study investigated whether memories formed in infanthood—during which it is known that animals exhibit amnesia for learned events—were still formed but in a dormant state unable to be recalled (i.e., infantile amnesia). By labeling the neurons active during the experience in infanthood and later artificially activating them in adulthood, these studies showed that latent memories could be artificially recalled, suggesting that the information relevant to the memory did indeed exist in the brain, but the animal had no natural way of recalling the memory in adulthood [92]. A subsequent study found that artificially activating this latent infantile memory in adulthood updated the memory, such that it could naturally be recalled thereafter [93]. This demonstrates that memories that are seemingly “lost” to infantile amnesia can be brought out of their latent state and be expressed as a typical long-term memory. The hippocampus is one of the few parts of the brain that experience neurogenesis in adulthood, and adult hippocampal neurogenesis has been proposed to support forgetting. Indeed, increasing neurogenesis enhances forgetting whereas decreasing neurogenesis leads memories to be better retained [94]. Sleep has also been proposed to be important for forgetting. For example, sleep has been shown to downregulate neuronal activity rates [95] as well as synapses [96], and this is thought to help support memory specificity [97] and forgetting. By globally downscaling synapses, this is thought to increase the signal-to-noise ratio of the system to remember the important things and discard the unimportant ones [96]. Another older theory was that rapid eye movement (REM) sleep, in particular, is involved in forgetting [98], which recent evidence supports [90].

### Schema learning

As we accumulate memories across our lives, we organize them into larger umbrellas of memories into which we categorize new memories. This has been termed schema learning. One breakthrough study found that after rats learned a hippocampus-dependent association task, it became easier for them to learn similar tasks thereafter. Critically, the subsequent learning was rapid and no longer dependent on the hippocampus. This study suggested that the way memories are consolidated depends on the prior history of the animal, and that consolidation can occur much more quickly if animals have prior history with similar experiences [99]. A recent perspective has described learning as a continuum as animals go from naïve to expert, and where animals are along this learning trajectory dictates which brain circuits are likely to be involved [100]. Animals frequently learn about structures and regularities in their external world, and one recent review distinguished among three similar yet distinct mechanisms of organizing spatial information in the brain: spatial schemas, cognitive maps, and spatial gists. Spatial schemas refer to a generalized representation of environments with similar spatial features, cognitive maps as allocentric representations of spatial relationships in a single environment, and spatial gists as the general and undetailed representation of a single environment. These different forms of memory organization are thought to be supported by distinct neural circuits, with cognitive maps and spatial gists being dependent on the hippocampus, while spatial schemas are independent of the hippocampus and instead dependent on cortical areas, consistent with Tse et al. [101]. How animals organize groups of memories as they accumulate related experiences is a topic of great interest and remains not well understood. Research on this topic will be critical in the service of understanding the richness of everyday memory, where memories are accumulated and related across a lifetime.

### Memory-linking

We experience the world continuously across time, and even when two memories that are formed adjacent in time have no relation to one another, we may be more likely to recall those two memories together than two memories that occurred distant in time to one another. How do we relate memories formed across time? A recent discovery that has provided insight into this question has been the phenomenon of memory-linking, which describes the process by which memories encoded close in time are integrated. In two of the seminal studies, it was discovered that mice link memories that are encoded within a few hours of each other. This memory-linking occurs because the neurons that are excitable to encode the first experience are also excitable to encode the second experience. By sharing an overlapping ensemble of neurons that encodes them, they become linked in memory [102, 103]. Another study showed that two different types of aversive memories (conditioned taste aversion and cued fear conditioning) can become linked when the two memories are recalled simultaneously. This simultaneous recall recruits a small subset of neurons—the overlapping ensemble across the two experiences—and this overlapping ensemble of neurons is critical for linking the two experiences, but not for representing either of them individually [104]. While the above studies have focused on how neuronal ensembles representing memories can be linked, a separate but related literature has focused on how synaptic plasticity at dendritic spines on the same neuron can drive memory-linking [105, 106]. For example, a recent preprint has demonstrated that memories that are formed within a day recruit activity in highly overlapping dendritic spines in the retrosplenial cortex, compared with memories formed 1 week apart [107]. This suggests that overlap in not only neuronal ensembles, but also dendritic compartments within a single neuron, could underlie the linking of memories across time. Interestingly, locus coeruleus-originating dopaminergic signaling to the hippocampus is necessary to drive memory-linking [108]. Memory-linking occurs when memories are encoded close in time, so what regulates the segregation of memories encoded across longer periods of time? A recent study demonstrated that CCR5, a chemokine receptor, has a delayed onset of expression following an experience, and its expression decreases neuronal excitability to prevent memory-linking across days [109]. This mechanism allows for memories that are experienced across days to be discriminated. However, we are capable of relating memories across longer periods of time than just hours, and how memories are integrated across longer periods of time is not as well understood [110]. One recent study demonstrated that a highly aversive experience can be linked with past neutral memories from days ago, and this form of memory-linking is driven by the offline reactivation of hippocampal ensembles representing the two memories; the ensembles representing the two memories are simultaneously reactivated (i.e., co-reactivated) thus driving the linking of the ensembles, and as a result, the linking of the memories (Zaki et al. [111]). Understanding how animals are flexibly able to relate experiences across seemingly arbitrary spans of time will be an important future direction in the field.

### Representational drift

**Fig. 2:**
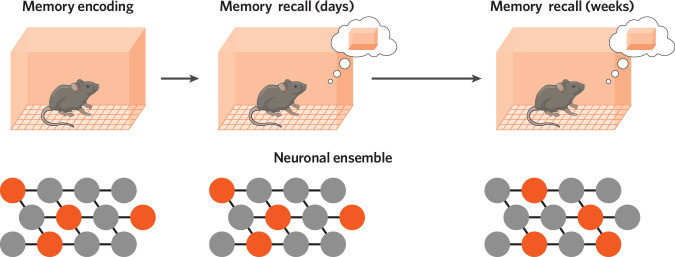
The memory ensemble is more dynamic than previously thought. Recent studies have challenged the view of the stable and permanent memory engram. Several studies have demonstrated that the neuronal activity patterns representing a stable memory can change over time, even though behavior remains stable. The change in neuronal activity patterns increases as a function of time despite the behavioral memory performance being stable. This poses a big question in the field of learning and memory- how is a stable memory stored in changing neural ensembles? How stable and dynamic are memory engrams?

Finally, a central question in neuroscience has been how the brain is flexible enough to actively learn about changes in the external world while remaining stable enough to retain memories of past experiences. While some studies have demonstrated that memories can be encoded in and represented by stable neuronal ensembles [16, 73–75, 92, 112, 113], recent studies have demonstrated that the neuronal activity patterns representing a stable memory can drift across time, even though behavior remains stable—termed representational drift (Fig. 2) [51, 114–118]. How, then, is a stable memory maintained if the neurons active during memory recall are constantly changing? This is an ongoing conversation in the field. Theories about the purpose of representational drift have been proposed, including that it is an inconvenient “bug” that the brain must compensate for, that this drift enables continual learning [51, 118–120], and that drift allows discrete experiences to be timestamped [121]. Some studies have demonstrated that while the individual neurons that are active during memory recall might drift across time, statistical features relevant to the memory are maintained in the neurons that are active [116], low-dimensional representations of behavior-relevant neural activity are maintained over time [117], and a full representation of a spatial environment is maintained by the active population [114]. Notably, two recent studies demonstrated that time and experience influence distinct features of representational drift in the hippocampus (i.e., rate coding and place field tuning of place cells, respectively) [122, 123]. In these studies, mice were trained to run along a one-dimensional track to receive water rewards on either end, and the tracks were highly familiar to the mice when the neural recordings were performed. As a result, there is no strong demand for the mice to use their memory to help guide their behavior. This has begged the question of whether drift occurs when a highly stable memory is not required. An elegant recent study demonstrated that, in the secondary motor cortex, neural representations of an olfactory association task drifted as mice acquired the task across days, but they were highly stable once the mice became expert performers [124]. This result suggests that the degree of representational drift could be related to the memory demand of the task. On the contrary, a separate study found that, in the piriform cortex, neural representations of odorants drifted across a month and that these representations were not stabilized when an odorant was associated with a footshock [125]. In other words, a strong memory for the odorant did not stop drift. One thought-provoking study suggested that representational drift is a consequence of not controlling for variations in behavior. By recording hippocampal activity in bats as they flexibly flew in a 3-dimensional environment, the authors found that neural representations of the environment did not drift and indeed were stable if the specific flight patterns were accounted for [126]. Notably, the studies described above have investigated different brain regions while animals are learning different tasks and performing different behaviors, and the readouts of a “neural representation” vary. On top of this, the recording modalities for measuring a neural representation vary, including technologies such as calcium imaging, electrophysiology, and IEG labeling and cell colocalization. Thus, it remains unclear whether and how representational drift varies as a function of these variables.

The observations of representational drift lend to the idea that while individual neurons representing a memory can drift, memory-relevant information can still be maintained in the population through population-level statistics. This provocative idea suggests that, in contrast to the idea of a stable engram, memories may not necessarily need to be represented in discrete sets of neurons; instead, which neurons are active at a given time to represent a memory can be flexible, so long as the overall population dynamics produce an output that a downstream brain area could interpret [31]. How the brain balances flexibility and stability to learn and retain memories across time remains an important question in neuroscience. We think an important direction for future research will be aimed at reconciling whether and how memories are stored in discrete or flexible neuronal populations.

## Conclusion

In the past century, we have made substantial progress in our understanding of memory. We now know that there are different forms of memory, including episodic, semantic, and procedural memory—which are all under the larger umbrella of long-term memory. There are also short-term forms of memory such as working memory. These memory systems are now known to be supported by different systems in the brain [127]. Memory has been studied extensively in different species, with attempts made to understand the common principles of memory across species [128, 129]. One incredible study demonstrated that a long-term associative memory could be formed in the mouse completely artificially in the absence of experience, by simultaneously stimulating known neural circuits representing conditioned and unconditioned stimuli in the brain [130]. Another remarkable study demonstrated, just this year, that high levels of neuronal activity during learning drives DNA damage, and DNA repair pathways are critical for learning—a cellular mechanism of learning that had not previously been appreciated [131]. These studies, among many others, are a true testament to the detailed understanding of learning and memory that has been accumulated in the last century. And yet there is still a significant amount that has yet to be discovered. How are memories organized across a lifetime? What biological principles predispose animals to have varying memory strengths of the same experience? How are memories in the brain chosen to be remembered or forgotten? How does the brain balance being stable enough to retain important information while remaining flexible enough to accommodate information about a changing external world [118]? Many questions remain in the study of memory.

A deeper understanding of the neurobiological basis of learning and memory has implications beyond just advancing basic science. Many psychiatric and neurological conditions are hallmarked by pathological memory function [9]. For example, post-traumatic stress disorder (PTSD) and certain anxiety disorders are thought to be memory-based psychiatric conditions where an aversive experience drives pathological fear in innocuous situations; Alzheimer’s disease and dementia are hallmarked by dramatic memory loss; and many patients who suffer from epilepsy struggle with memory problems as well. By understanding how memory systems function in neurotypical conditions, we will have a stronger grasp of how these memory systems go awry in pathological cases. The basic study of memory in the past century has provided us with a rich understanding of how memories are encoded, consolidated, and recalled. More recent work has shed light on how memories can be updated, integrated, and segregated across time and experience. Ultimately, future work aimed at understanding how memories are dynamically updated across the lifespan will inform both how memory systems function in health as well as in disease.
